# Supplementary material for: Resolving the full spectrum of human genome variation using Linked-Reads
Source: Genome Res. 2019 Apr;29(4):635–45. doi: 10.1101/gr.234443.118 (PMC6442396; doi:10.1101/gr.234443.118)
Supplement: Supplemental Material [file supp_gr.234443.118_Supplemental_longranger-2.2.zip › longranger-2.2.tar.bz2!/lr2.2-public/lib/stan/cmdstan/stan/doc/api/html/agrad_2fwd_2matrix_2multiply__lower__tri__self__transpose_8hpp_source.html]

Stan: src/stan/agrad/fwd/matrix/multiply\_lower\_tri\_self\_transpose.hpp Source File


|  |  |
| --- | --- |
|  | Stan  2.5.0  probability, sampling & optimization |


- Main Page
- Related Pages
- Namespaces
- Classes
- Files

- File List
- File Members

All Classes Namespaces Files Functions Variables Typedefs Enumerations Enumerator Friends Macros Pages

- src
- stan
- agrad
- fwd
- matrix

multiply\_lower\_tri\_self\_transpose.hpp

Go to the documentation of this file.

1 #ifndef STAN\_\_AGRAD\_\_FWD\_\_MATRIX\_\_MULTIPLY\_LOWER\_TRI\_SELF\_TRANSPOSE\_HPP

2 #define STAN\_\_AGRAD\_\_FWD\_\_MATRIX\_\_MULTIPLY\_LOWER\_TRI\_SELF\_TRANSPOSE\_HPP

3

4 #include <vector>

5 #include <stan/math/matrix/Eigen.hpp>

6 #include <stan/math/matrix/typedefs.hpp>

7 #include <stan/agrad/fwd/matrix/typedefs.hpp>

8 #include <stan/agrad/fwd/matrix/multiply.hpp>

9 #include <stan/math/matrix/transpose.hpp>

10

11 namespace stan {

12  namespace agrad {

13

14  template<typename T,int R, int C>

15  inline

16  Eigen::Matrix<fvar<T>,R,R>

17  multiply\_lower\_tri\_self\_transpose(const Eigen::Matrix<fvar<T>, R, C>& m) {

18  if (m.rows() == 0)

19  return Eigen::Matrix<fvar<T>,R,R>(0,0);

20  Eigen::Matrix<fvar<T>,R,C> L(m.rows(),m.cols());

21  L.setZero();

22

23  for(size\_type i = 0; i < m.rows(); i++) {

24  for(size\_type j = 0; (j < i + 1) && (j < m.cols()); j++)

25  L(i,j) = m(i,j);

26  }

27

28  return stan::agrad::multiply(L, stan::math::transpose(L));

29  }

30

31  }

32 }

33 #endif

typedefs.hpp

stan::agrad::fvar

**Definition:** fvar.hpp:13

typedefs.hpp

stan::agrad::multiply

Eigen::Matrix< fvar< T >, R1, C1 > multiply(const Eigen::Matrix< fvar< T >, R1, C1 > &m, const fvar< T > &c)

**Definition:** multiply.hpp:21

transpose.hpp

stan::math::transpose

Eigen::Matrix< T, C, R > transpose(const Eigen::Matrix< T, R, C > &m)

**Definition:** transpose.hpp:12

stan::agrad::size\_type

Eigen::Matrix< double, Eigen::Dynamic, Eigen::Dynamic >::Index size\_type

**Definition:** typedefs.hpp:14

Eigen.hpp

multiply.hpp

stan::agrad::multiply\_lower\_tri\_self\_transpose

Eigen::Matrix< fvar< T >, R, R > multiply\_lower\_tri\_self\_transpose(const Eigen::Matrix< fvar< T >, R, C > &m)

**Definition:** multiply\_lower\_tri\_self\_transpose.hpp:17


---

[ Stan Home Page ]

*© 2011–2014,
Stan Development Team.*
